# Supplementary material for: Plant genotype influence the structure of cereal seed fungal microbiome
Source: Front Microbiol. 2023 Jan 4;13:1075399. doi: 10.3389/fmicb.2022.1075399 (PMC9846234; doi:10.3389/fmicb.2022.1075399)
Supplement: Supplementary file 1 [file Data_Sheet_1.docx]

**Supplementary material**

**Table S1.** Pairwise post-hoc PERMANOVA contrasts between plant species (FDR-corrected). P-values in bold are < 0.05.

| **Contrast** | **p-value** |
| --- | --- |
| *A. sativa – H. vulgare* | **0.0300** |
| *A. sativa – S. cereale* | **0.0300** |
| *A. sativa – T. aestivum* | **0.0075** |
| *A. sativa – T. polonicum* | 0.0600 |
| *A. sativa – T. turgidum* | **0.0075** |
| *H. vulgare – S. cereale* | 0.6130 |
| *H. vulgare – T. aestivum* | **0.0300** |
| *H. vulgare – T. polonicum* | 0.0575 |
| *H. vulgare – T. turgidum* | 0.4080 |
| *S. cereale – T. aestivum* | 0.5862 |
| *S. cereale – T. polonicum* | 0.4080 |
| *S. cereale – T. turgidum* | 0.6130 |
| *T. aestivum – T. polonicum* | 0.4718 |
| *T. aestivum – T. turgidum* | 0.0862 |
| *T. polonicum – T. turgidum* | 0.5862 |

**Figure S1**. Relative abundance of fungal genera (>1%) for each host plant species.
